# Self-Tuning Method for Increased Obstacle Detection Reliability Based on Internet of Things LiDAR Sensor Models

**DOI:** 10.3390/s18051508

**Published:** 2018-05-10

**Authors:** Fernando Castaño, Gerardo Beruvides, Alberto Villalonga, Rodolfo E. Haber

**Affiliations:** 1 Centre for Automation and Robotics, UPM—CSIC, 28500 Arganda del Rey, Spain; gerardo.beruvides@car.upm-csic.es (G.B.); alberto.villalonga@umcc.cu (A.V.); rodolfo.haber@car.upm-csic.es (R.E.H.); 2 Research Centre of Advanced and Sustainable Manufacturing, UM, Matanzas 44100, Cuba

**Keywords:** LiDAR sensors reliability, Internet of Things, self-turning parameterization, k-nearest neighbors, driven-assistance simulator

## Abstract

On-chip LiDAR sensors for vehicle collision avoidance are a rapidly expanding area of research and development. The assessment of reliable obstacle detection using data collected by LiDAR sensors has become a key issue that the scientific community is actively exploring. The design of a self-tuning methodology and its implementation are presented in this paper, to maximize the reliability of LiDAR sensors network for obstacle detection in the ‘Internet of Things’ (IoT) mobility scenarios. The Webots Automobile 3D simulation tool for emulating sensor interaction in complex driving environments is selected in order to achieve that objective. Furthermore, a model-based framework is defined that employs a point-cloud clustering technique, and an error-based prediction model library that is composed of a multilayer perceptron neural network, and k-nearest neighbors and linear regression models. Finally, a reinforcement learning technique, specifically a Q-learning method, is implemented to determine the number of LiDAR sensors that are required to increase sensor reliability for obstacle localization tasks. In addition, a IoT driving assistance user scenario, connecting a five LiDAR sensor network is designed and implemented to validate the accuracy of the computational intelligence-based framework. The results demonstrated that the self-tuning method is an appropriate strategy to increase the reliability of the sensor network while minimizing detection thresholds.

## 1. Introduction

Nowadays, Internet of Things (IoT) applications are present in many sectors that range from industrial environments (e.g., manufacturing, energy, etc.) to our personal lives (e.g., health, society, mobility, etc.). The IoT is a strategic innovation in automotive applications that has received support and investment in recent years, in order to develop smart mobility ecosystems for the market with an autonomous level of interaction between vehicles and infrastructures. Nevertheless, mainstream car manufacturers, OEMs for the automotive sector, researchers, and engineers are introducing new technological contributions as new short-term challenges have to be addressed [1,2].

One particular challenge of autonomous driving is the accuracy and reliability estimation in vision devices such as light detection and ranging (LiDAR) sensors and stereo cameras integrated in automotive driving assistance systems for pattern recognition and obstacle detection tasks [3]. In many scenarios, it is very difficult to certify the real topology and distance of objects at a lower level of uncertainty, in most cases due to dead zones, object transparency, light reflection, weather conditions, and sensor failures, among others [4]. Furthermore, traditional networking devices are not designed for use in unpredictable, variable, and dynamic environments that characterize an IoT transportation ecosystem, making it necessary to develop new methodologies that characterize and estimate sensor reliability [5]. Sensor fusion is commonly applied to combine different sensors for road detection, mainly cameras and LiDARs. Current sensor fusion methods are even taking advantage of both types of sensors (cameras or LiDARs), rather than exploiting the advantages of each individual type of sensor [6]. Furthermore, the parallel processing of frames (from a camera) and (LiDAR) scans implies a high computational cost and is unnecessary in many scenarios where a sensor-based error-prediction model for assessing runtime reliability is operative [7].

Another important issue is the increase of computing power and wireless communication capabilities to expand the role of sensors from mere data collection to more demanding tasks that include sensor fusion, classification, and collaborative target tracking. Fault tolerance and reliability perform a key role for embedded systems, such as obscured wireless sensors that are deployed in applications where physical access is difficult [8]. Reliable monitoring of a phenomenon (or event detection) depends on the set of data provided by the cluster of sensors rather than any individual node. The failure of one or more nodes may not cause the disconnection of operational data sources from the data sinks (command nodes or end user stations). However, node failures may increase the number of hops a data message has to go through before reaching its destination (thereby increasing the message delay), providing an estimation of the failure probabilities of both the sensors and the intermediate nodes (nodes used to relay messages between data sources, and data sinks) [9].

Several reconstruction methods are reported in the literature to create specific geometric models of existing objects from scanned point clouds based on information obtained from LiDARs [10]. Progress with modelling techniques that simulate complex driving environments has led to realistic representations of multiple input/output variables, which have been used to determine the most influential factors in degraded reliability and to detect pedestrians, obstacles, and vehicles in real-time driving scenarios [11]. Clustering techniques are intensively used in exploratory data mining, statistical analysis, pattern recognition, image analysis, information retrieval, bioinformatics, data compression, and computer graphics [12]. One widely-used clustering algorithm is the *k*-nearest neighbor (*k*NN) algorithm, due to its effectiveness at isolating the nearest neighbors of a query in a training dataset and then predicting the query with the major class of nearest neighbors [13]. Another widely applied technique in industrial applications is reinforcement learning [14]. Exemplary results have been achieved with the Q-learning algorithm [15], which generates artificial intelligence and self-learning strategies in complex processes and that has a self-tuning capability to obtain the optimal configuration, based on rewards or penalties learned in previous states (iterative knowledge generation) [16].

The main contributions of this work are the design a four-step method and its implementation to maximize the reliability of an IoT LiDAR sensor network and to minimize the detection threshold (*γ*, the number of LiDAR sensors required to detect one obstacle). The method includes point cloud grouping, modeling, learning, and self-tuning (knowledge-based learning algorithm) tasks, combining supervised and reinforcement machine learning techniques and clustering. Furthermore, an IoT driving assistance scenario with a sensor network is created using the Webots simulation tool to generate a LiDAR scan benchmark. Finally, the method is validated in a dynamic obstacle detention scenario, in order to obtain the best prediction model and the optimal number of LiDAR sensors needed to guarantee obstacle localization reliability. Indeed, networked control systems and Internet of Things are key topics for the next generation of automated driving systems. Nevertheless, this paper does not deal with networked control systems. This paper is focused on IoT LiDAR sensors. Computational intelligence methods are then applied to determine the number of LiDAR sensors that are required to increase sensor reliability for obstacle localization tasks. Further studies related with the application of network-based fuzzy control and H_∞_ control strategy to automated driving systems will be explored in the future [17,18].

The paper consists of five sections. Following this introduction, the second section will show the design and the implementation of several modules that employ self-tuning methods for improved reliability. Subsequently, a driving-assistance case study scenario for obstacle detection based on IoT LiDAR model sensor information will be developed in Section 3. Additionally, the proposed methodology will be validated with the minimum required number of sensors, to ensure LiDAR sensor reliability in each scan. Finally, the conclusions and future research steps are presented.

## 2. Self-Tuning Method for Reliability in LiDAR Sensors Network

The self-tuning method for reliability in LiDAR sensor networks mainly consists of a computer-aided system that enables efficient data interchange between the data transmitted from the IoT sensor networks, managed by a control node network, and external modules that evaluate network behavior. The supervisor node controller (SNC) is the component in charge of generating sensory data through the simulation of sensor models; while the IoT assessment framework is the interface with the external modules. Different simulation tools can be used for this purpose. On the one hand, there is a 3D model simulator for automotive applications and on the other hand, an external programming software with a set of toolkits to manage points cloud, clustering methods, pattern recognition algorithms, and artificial intelligence (AI) based modelling strategies, among others.

### 2.1. Conceptual Design

**Figure 1:**
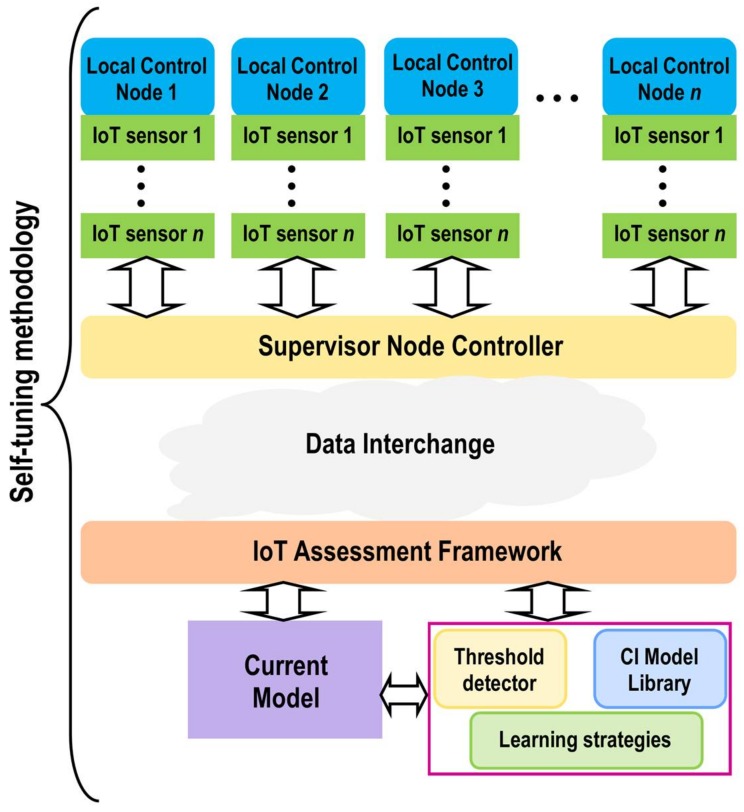
Conceptual design of self-tuning method. Iteration between IoT assessment framework and supervisor node controller.

The conceptual and architectural design of the proposed method is presented in this section. Figure 1 shows the data interchange model between sensory nodes and actuation. The data interchange component operates as data-sharing broker. The SNC and the IoT assessment framework input information into the data interchange broker and collect information from it.

The SNC comprises different local control nodes containing IoT sensor network models, distributed according to their functions. The distributed IoT sensors are in charge of capturing sensory data and interchange these data with the SNC, in order to share it with other external modules. It is important to highlight that data must necessarily pass through the supervisor, when information is sent to and received from the different nodes of the IoT sensors network. However, the same procedure is not necessary when this data transfer is between the different IoT sensors that comprise the sensor network.

The IoT assessment framework sends/receives data to/from the SNC. The first key component is a module developed for a customized function directly linked to the local control nodes and the IoT sensor network. The training procedure is performed by using classification methods based on computational intelligence techniques such as *k*-nearest neighbors, multi-layer perceptron, support vector machines, self-organizing map, Bayesian networks, etc. For example, on the one hand, a module can be in charge of predicting the error in the localization of an object from data cloud points given by a LiDAR sensor. On the other, it can involve a group of tasks for self-tuning (knowledge-based learning algorithm). This module consists of a computational intelligence (CI) library of models that contains various models with similar functions and a learning strategy (i.e., Q-learning) that computes the actual threshold value in runtime, for the implementation of corrective actions. Both methods can also be enriched at runtime with data received from the nodes of the IoT sensor network.

### 2.2. Implementation

The SNC, the local control nodes and IoT sensor network are designed and implemented using a simulation tool for 3D models using Webots R2018a for automobiles [19]. In addition to its high degree of potentiality when simulating sensors for driving assistance, Webots is able to interact with other external software or programming languages, such as MATLAB, Python, Java, and Visual #C/C++, among others. It should be noted that for modelling and simulation of sensors, any other simulation tool for 3D sensor models can be selected.

An IoT assessment framework can therefore be implemented using any of the previously mentioned software or external programming languages. One of these software programming languages with an extensive set of libraries, MATLAB 2017b, was selected for developing the self-tuning procedure. These tasks are carried out by two parallel execution threads, one of which is in local mode with direct data transfer from the IoT sensor network, while the other functions at a global level. The local thread (parallel execution 1) executes the current error prediction model from sensory data provided by IoT sensor network. Subsequently, depending on the value of a certain threshold that is calculated in runtime through a learning process, a set of corrective actions are performed. This procedure is described in later sections.

On the other hand, the global thread (parallel execution 2) contains the CI model library with other error prediction models and different performance indices. Later on, the library can also enriched with the process simulation. During the simulation, new sensory data can be generated providing new information on the environment in each interaction. Based on this continuous information flow and the previous knowledge-based learning algorithm, the library executes a parallel learning procedure for all error prediction models, to obtain a personalized setting for each particular critical situation. Finally, once a new best configuration is generated, the corresponding model in the IoT sensor network is then updated.

#### 2.2.1. Supervisor Node Controller

The supervisor node controller manages the scenario in runtime and interchange data between local control nodes or IoT sensors with other external modules. The overall operation of the 3D scenario is managed by the SNC, in this case, Webots. Webots origins can be traced back to an extension of robotic simulation software adapted to automobile simulations in a virtual environment. A set of computational procedures manages the adaptation and transference of sensory information. The data transfer is performed by means of different functionalities available in Webots. For example, some available functions serve to create sensor models, such as LiDARs, stereo vision cameras, radar, inertial, magnetic, gyroscopic, and GPS sensors that can be emulated with this software. In addition, many obstacles and objects can be added to the scenario, such as simple vehicles, road segments, traffic signals and lights, buildings, etc. A 3D traffic scenario can therefore be created, in order to simulate the behavior of IoT sensor networks, which is incorporated in each local control node (i.e., a fully automated vehicle) for driving-assistance scenarios.

#### 2.2.2. Computational Intelligence Library for Modeling

The library of computational intelligence methods comprises three widely used techniques to identify dynamic ecosystem patterns and for their predictive modeling. First, a multi-layer perceptron (MLP) artificial neural network was selected. MLP is the most widely applied topology as a universal approximator, guaranteeing higher performance with only one hidden layer and therefore enabling good modeling capabilities [20,21,22]. The MLP training process is based on the error back-propagation algorithm. It is a supervised learning rule performed in two iterative steps. First, the weights and biases are randomly initialized. Subsequently, the training samples are presented one at a time. Then, for each *s*-th sample, the output of each neuron is computed from its inputs

(1) $$y_{j}^{\left(r\right)}(s)=f[u_{j}^{\left(r\right)}(s)]$$

which may be expressed as

(2) $$u_{j}^{\left(r\right)}(s)=\sum_{i=0}^{R^{\left(r-1\right)}}w_{i,j}^{\left(r\right)}x_{i}^{\left(r\right)}(s)+b_{j}^{\left(r\right)}$$

where, *f* (•) is the activation function; *R* is the total number of layers; $y_{j}^{\left(r\right)}(s)$, the output of the *j*-th neuron in the *r*-th layer for the *s*-th sample; $b_{j}^{\left(r\right)}$, the bias of this neuron; $w_{i,j}^{\left(r\right)}$, the weight connecting the *i*-th neuron in the (*r* − 1)-th layer with the *j*-th neuron in the *r*-th layer; and $x_{i}^{\left(r\right)}(s)$ is the input from the *i*-th neuron in the (*r* − 1)-th layer for the *s*-th sample.

The second selected technique was a *k*-nearest neighbors (*k*NN) clustering algorithm. *k*NN is one of the simplest non-parametric classification methods with an easily interpretable output, low calculation times, and high predictive power. Furthermore, it is widely used in classification and regression problems for real applications [23,24,25,26]. The algorithm is based on the predictions for a new instance (*x*) by searching through the entire training set for the most similar instances of *k* (the neighbors) and summarizing the output variable for those *k* instances. A Euclidean distance (ED) is determined, to select the *k* instances in the training dataset that are the most similar to a new input.

(3) $$ED(x,x_{i})=\sqrt{\sum_{j=1}^{n}{\left(x_{j}-x_{ij}\right)}^{2}}$$

The *k*NN algorithm is executed by following the five steps listed below:

- Calculate the distance between test data and each row of training data
- Sort the calculated distances in ascending order based on distance values
- Select the top *k* rows from the sorted array
- Select the most frequent class of these rows
- Return the predicted class

Finally, a multiple linear regression technique was implemented to adjust the obstacle localization model. The model can be represented as

(4) $$y^{\left(P\right)}=b^{T}x=[b_{0},\;b_{1},\;\ldots ,\;b_{n}]\left[\begin{array}{c} 1\\ x_{1}\\ M\\ x_{n} \end{array}\right]$$

relating some input variables, *x*, with an output, *y*. Coefficients, *b*, are obtained by minimizing the sum of the square of the difference between the predicted and the observed values, $y^{\left(P\right)}$ and y, for a set of m input-output pairs

(5) $$X=\left[\begin{array}{cccc} 1 & x_{11} & \ldots & x_{1m}\\ \vdots & \vdots & & \vdots \\ 1 & x_{n1} & \ldots & x_{nm} \end{array}\right];\;y=\left[\begin{array}{c} y_{1}\\ \vdots \\ y_{n} \end{array}\right]$$

the regression coefficients can be estimated through

(6) $$b={\left(X^{T}X\right)}^{-1}X^{T}y$$

#### 2.2.3. Threshold Detector and Q-Learning Procedure

The Q-learning algorithm is a model-free reinforcement learning technique. Specifically, Q-learning can be used to find an optimal action-selection policy for any given (finite) Markov decision process [27,28]. It learns an action-value function that ultimately yields the expected utility of taking a given action in a given state and it then follows the optimal policy (off-policy learner). The algorithm is based on a simple value iteration update. It assumes the old value and introduces a correction based on the new information [29].

(7) $$Q(s_{t+1},a_{t+1})\leftarrow Q(s_{t},a_{t})+\alpha \cdot (r_{t+1}+\gamma \cdot \underset{a\in A}{\max}Q(s_{t+1},a_{t})-Q(s_{t},a_{t}))$$

where, *r_t_*_+1_ is the reward observed after performing *a_t_* in $s_{t}$, and *α* is the learning rate (0 < *α* ≤ 1).

**Figure 2:**
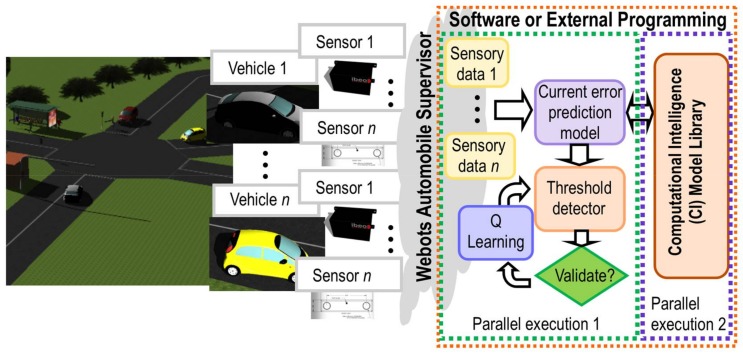
Implementation of self-tuning procedure (knowledge-based learning algorithm).

The Q-learning algorithm is introduced in the closed-loop cycle (self-tuning), in order to minimize the numbers of LiDAR sensors needed to guarantee good accuracy in the localization of a detected obstacle within a minimum computation time, as illustrated in Figure 2. Table 1 shows different rewards assigned for each detection threshold (*γ*).

This range of values is defined as a function of the obstacle prediction error calculated during the classification step (see Figure 2). During the learning process, the algorithm recommends the optimal numbers of sensors to achieve a reliable obstacle detection, based on the previous knowledge generated by the rewards obtained from similar situations learnt in the past. This self-turning threshold produces a reduction of the computational cost and accelerates the prediction time that is needed to detect an obstacle in the driving assistance environments.

## 3. IoT LiDAR Sensor Models for Obstacle Detection—A Case Study

**Figure 3:**
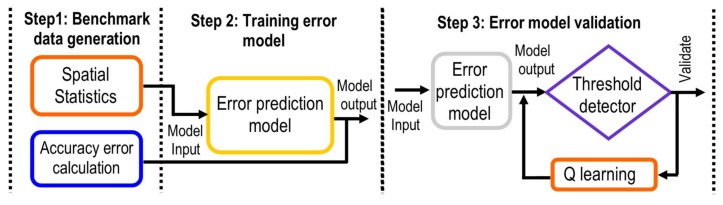
Overall scheme of the methodology of the self-tuning method.

A particular driving assistance scenario is defined, in order to evaluate the proposed self-tuning methodology for its validation. In this use case, the methodology was applied to a LiDAR sensor model to assess the reliability related to the error accuracy in the location of an object. In this section, a model of error prediction from data of a single LiDAR sensor model was generated. Instead, this same model will be used in a later section to evaluate and to establish the reliability of an IoT sensor network. The method of generating a dataset for training and to validate the error prediction models is described in the following sections (see Figure 3).

### 3.1. Training Dataset from 3D Scenario Simulation

**Figure 4:**
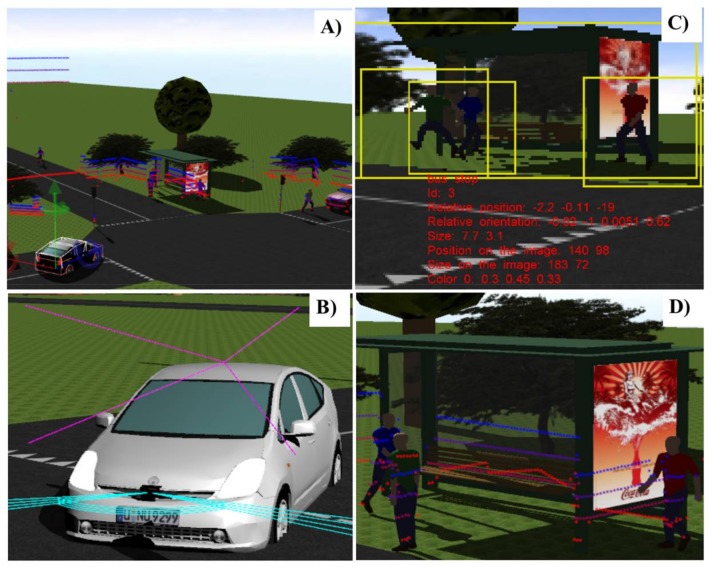
(**a**) Aerial view of simulated 3D scenario in Webots automobile; (**b**) Vehicle model with sensors incorporated; (**c**) Image captured by the camera and object detection procedure; (**d**) Point cloud projection from the LiDAR sensor over objects once detected.

The Webots automobile simulation tool in conjunction with a four-layer LiDAR sensor model was used, in order to generate a virtual driving traffic scenario. The scenario emulates the real test track (a roundabout, traffic lights at a central crossroad and additional curves on an otherwise mainly straight track) at the Centre for Automation and Robotics (CAR) in Ctra. Campo Real Km. 0.2, Arganda del Rey (Madrid, Spain), which simulates an urban environment with pedestrians and a fleet of six fully-automated vehicles in movement and a communications tower. A more detailed description of this scenario and both the real and simulated parts can be found in [30]. Figure 4 illustrates the aerial view of some of these 3D scenarios in Webots Automobile for driving assistance. A vehicle model (Toyota Prius), a camera image with objects that have been recognized and the LiDAR point cloud are also illustrated in Figure 4. The objective of this camera is to obtain high-precision object location for comparison with the location obtained by the LiDAR sensor, thereby generating the error prediction models.

The fully sensorized vehicle model (Toyota Prius model) incorporated two on-board sensors, one LiDAR sensor, and a 3D stereo vision camera (see Figure 4b). Both sensors were positioned inside the vehicle, the LiDAR in the lower front section and the camera in the upper front section. Table 2 shows the specifications and localization of this sensor mounted on the vehicle.

#### 3.1.1. Benchmark Data

Following a simulation of 2 min and 56 s, data collection provided by the LiDAR sensor model and a set of images captured by the 3D stereo vision camera were obtained. In total, a benchmark of 1031 scenes was available with the same number of LiDAR scans, captured images, and annotation files with the localization of each recognized obstacle.

On the one hand, the camera benchmark contained 1031 images and their corresponding annotation files, obtained from the camera-sensor object recognition algorithm, with the following information: the localization (weight × height) of each object in the image (in pixels) and the size (weight x height) of each object in the image. This sensor is a stereo vision camera with the following specifications: 0.8 MP; resolution 1032 × 776, color, and 20 FPS.

On the other hand, the LiDAR benchmark contained 1031 scans. Each scan contained a 3-D point cloud/scan. This small benchmark set was useful for exploring the accuracy of an obstacle in the scene. Each scene contained the relative *X*, *Y*, *Z* position of an object in the first, the second, and the third columns, showing the LiDAR localization coordinates at the scene (*X*_0_ = 0, *Y*_0_ = 0 and *Z*_0_ = 0), and the fourth column listed the number of the corresponding layer.

In addition, the raw data from the LiDAR sensors required filtering and pre-processing in order to facilitate determination of the location error of the object. Firstly, the points that map the road asphalt at ground level and the vegetation were deleted. These points were mainly located at 20 cm above ground level.

Secondly, fast indexing and search capabilities were required, in order to process the sensory data. To do so, the point cloud was internally organized using a k-d tree structure; a space partitioning data structure for mapping points onto a k-dimensional space [31]. The next data-processing step consisted of extracting the points by mapping nearby obstacles that correspond to a specific point-cloud sequence. A density-based spatial clustering of applications with a noise (DBSCAN) algorithm was applied [32] for segmentation, which can segment the point cloud for each available obstacle at the scene. The algorithm generated the points as clusters around each axis, and Equation (7) was used to calculate the centroid of each segmented (*X*_0_, *Y*_0_, *Z*_0_) point cloud that corresponded to each obstacle.

The final step was to compare each LiDAR calculated centroid with the actual location generated by the object recognition algorithm of each obstacle, in order to obtain the accuracy error.

Once the benchmark data set had been created, the next step was to generate the training data set for the generation of the error prediction models. The spatial statistics of the point cloud were then used as inputs for the error prediction model. Subsequently, the two errors that were taken into account as outputs of the model were also explained.

#### 3.1.2. Model Inputs

A group of spatial statistics were implemented, in order to standardize the model inputs independently from the point cloud distribution [33]. The centrographic and directional distribution of the point clouds were obtained from these spatial point pattern methods. Common centrographic statistics for a point pattern are as follows: mean center, median center, standard deviational circle, and standard deviational ellipse [34]. The mean center, *MC*, is characterized by geographic coordinates {*X*, *Y*, *Z*} equal to the arithmetic means of the *x*-, *y*-, and *z*-coordinates of all the *N* points in a pattern

(8) $$MC(X)=\frac{\sum_{i=1}^{N}x_{i}}{N};\;MC(Y)=\frac{\sum_{i=1}^{N}y_{i}}{N};\;MC(Z)=\frac{\sum_{i=1}^{N}z_{i}}{N}$$

An alternative unique measure of the central spatial tendency of a point pattern that was used is the center of minimum distance (often referred to as the median center), which is robust in the presence of spatial outliers. Unlike the mean center, defining the median center, *MedC*, requires a much more computationally complex iterative process to find a location that minimizes the Euclidean distance, d, to all the points in a point pattern [35]

(9) $$MedC\left\{x^{t},y^{t},z^{t}\right\}|\min\left(\sum_{i=1,t=1}^{i=N,t=A}\sqrt{{\left(x_{i}-x^{t}\right)}^{2}+{\left(y_{i}-y^{t}\right)}^{2}+{\left(z_{i}-z^{t}\right)}^{2}}\right)$$

where, *i* defines each point in a point pattern, *t* is an iteration number, and {*x^t^*, *y^t^*, *z^t^*} is a location of the median center of an iterative candidate. An important property of a point pattern is the degree of its spatial spread. It can be characterized by the standard distance, *SD*, that is estimated as

(10) $$SD=\sqrt{\frac{\sum_{i=1}^{n}{\left(x_{i}-MC(X)\right)}^{2}}{N}+\frac{\sum_{i=1}^{n}{\left(x_{i}-MC(Y)\right)}^{2}}{N}+\frac{\sum_{i=1}^{n}{\left(x_{i}-MC(Z)\right)}^{2}}{N}}$$

where, *x_i_*, *y_i_*, and *z_i_* are the coordinates of point *i*{*x_i_*, *y_i_*, *z_i_*}, *N* is the total number of points, and *MC*(*X*), *MC*(*Y*), and *MC*(*Z*) are the coordinates of the mean center. The standard distance that results from the different average distances to a given centroid is usually graphically represented in a geographic information system (GIS) environment by a standard deviational circle, centered on the mean center with the radius equal to the standard distance.

Finally, the last spatial statistic used as inputs in this work is the third central moment (3rd*CM*) [36]. This value represents the mean value of the cubic deviation in distance from each point to the *MC* of each axis. The 3rd*CM* value is calculated as

(11) $$3\mathrm{rd}CM=\frac{1}{n}\sum_{i=1}^{n}\left[{\left(x_{i}-MC(X)\right)}^{2}+{\left(y_{i}-MC(Y)\right)}^{2}+{\left(z_{i}-MC(Z)\right)}^{2}\right]$$

where, *x_i_*, *y_i_*, and *z_i_* are the coordinates of point *i*{*x_i_*, *y_i_*, *z_i_*}, *n* is the total number of points and the *MC* is the mean center by geographic coordinates {*X*, *Y*, *Z*}, as calculated in Equation (3).

#### 3.1.3. Model Output

The outputs of these models are two figures of merit of accuracy: the distance root mean squared (DRMS) and the mean radial spherical error (MRSE). The first is a measure of data tracked on the *x*–*y* plane (2D) and the second is a measure of the data tracked in an *x*–*y*–*z* space (3D) [37]. The DRMS and MRSE values were calculated as

(12) $$DRMS=\sqrt{\frac{1}{n}\sum_{i=1}^{n}\left\{{\left(x_{i}-x_{Actual,ti}\right)}^{2}+{\left(y_{i}-y_{Actual,ti}\right)}^{2}\right\}}$$

(13) $$MRSE=\sqrt{\frac{1}{n}\sum_{i=1}^{n}\left\{{\left(x_{i}-x_{Actual,ti}\right)}^{2}+{\left(y_{i}-y_{Actual,ti}\right)}^{2}+{\left(z_{i}-z_{Actual,ti}\right)}^{2}\right\}}$$

where, *n* is the number of readings for a dynamic tag during the time it is tracked, (*x_ti_*, *y_ti_*, *z_ti_*) are the coordinates of the tag at time *t_i_*, and (*x_Actual,ti_*, *y_Actual,ti_*, *z_Actual,ti_*) are the actual coordinates of the tag at time *t_i_*.

### 3.2. Model Training and Initial Validation

The error-based prediction model library for the localization is defined, in order to estimate the value of the figure of merits in terms of error (DRMS and MRSE), as a function of the parameters extracted from the point cloud generated by the LiDAR sensors. Three models were considered in this approach. First, a multilayer perceptron neural network with backpropagation (MLP) comprising two hidden layers, with five neurons for each hidden layer, sigmoid activation functions, and 10^4^ epochs were trained. The learning rate (*μ*) initial value was 10^−3^ with a decrease factor ratio of 10^−1^, an increase factor ratio of 10, and a maximum *μ* value of 10^10^. The minimum performance gradient was 10^−7^. The adaptive value *μ* is increased by 10 until the change above results in a reduced performance value. The change is then made to the network and *μ* is decreased by 10^−1^. Training stops when any of these conditions occurs as follows: the maximum number of epochs (repetitions) is reached, or the maximum amount of time is exceeded, or the performance is minimized to the goal, or the performance gradient falls below minimum gradient, or *μ* exceeds 10^10^.

The second modeling technique was a two-neighbor *k*-nearest neighbors (*k*NN). Finally, a lineal regression was also obtained by minimizing the sum of squared differences between the predicted and the observed values.

A total of 1031 scans were extracted from the Webots simulator to generate the training and the validation datasets. Subsequently, the scans were randomly divided into two datasets: 765 samples for the training dataset (representing the 74% of the total of samples) and 255 samples to compose the validation dataset (representing the other 26% of the total of all samples). The model correlation coefficients (R^2^) were estimated for all the models implemented in the modeling library.

Table 3 showed the values obtained for each model based on the plane (DMRS) and the space (MRSE) figures of merits described previously. As can be seen, the *k*NN algorithms represent, in both cases, the best fitting parameters, with a 93% correlation between the *x*–*y*–*z* cloud of points coordinates in the localization of each obstacle that was detected.

**Figure 5:**
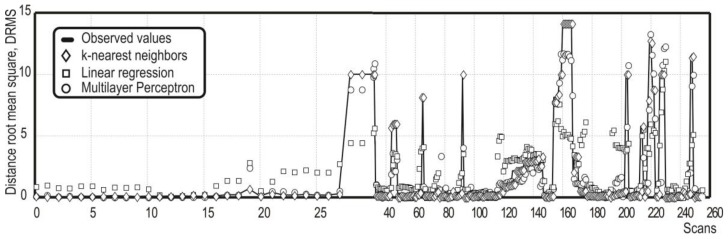
Prediction error behavior of the model library in the localization of obstacles by LiDAR point clouds.

Finally, the DRMS tendency for all the models is shown in Figure 5, validating the best fit obtained between the observed solution and the prediction values based on the *k*NN algorithm. Nevertheless, in most cases, the MLP model presented a very similar behavior with the *k*NN model, and not so very different from the linear regression model.

## 4. Experimental Results

Additional experimental tests were conducted for the evaluation of the IoT on-chip LiDAR sensor network performance and the self-tuning methodology performance in the dynamic obstacle detention scenario. The aim was to define the best prediction model and the optimal number of LiDAR sensors required to ensure the reliability of this sensor network. A set of critical conditions were taken into account in the study.

In this case, the simulation time (43 s) for each traffic scenario was somewhat smaller, due to the computational overload generated by the processing and storage of all the data provided by five LiDARs plus two high-resolution cameras. In this experimental evaluation, the 3D stereo vision was only activated when all the sensors, including the LiDAR sensors, were incapable of guaranteeing sufficient reliability in terms of safety. In addition, the global scenario was the same as the one described in Section 3. However, there are plenty of devices and objects in each scene with different distributions. Some of these dynamic objects in the scenario are 4 buildings, 50 trees, 20 pedestrians, 10 small and medium vehicles, and 1 truck. Therefore, the main difference relies on the distribution of the IoT sensory system in the fully automated vehicle (Toyota Prius) modelled in Webots (see Figure 5).

**Figure 6:**
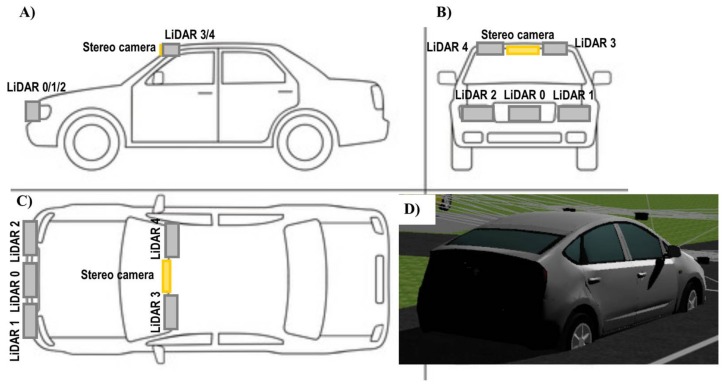
Side (**a**); front (**b**); plan (**c**) and rear view (**d**) of on-board IoT sensory system setup in a vehicle model.

Figure 6 represents the configuration of the IoT sensory system mounted in a fully automated vehicle modeled in a Webots automobile.

Three equidistant LiDAR models (LiDAR 0/1/2) were attached at a low level on the front of the vehicle, in order to expand the horizontal field of view. Two LiDAR devices (LiDAR 3/4) were placed at a higher level on the front of the vehicle, on either side of the stereoscopic camera model, the purpose of which is, in this case, to expand the vertical field of view. The aim of this IoT evaluation is to demonstrate how an IoT sensory system incorporates a series of extended precision-related measurement capabilities, with regard to the behavior of a single sensor with better specifications than each isolated node of the sensory network. The setup of the IoT sensory system is summarized in Table 4.

**Figure 7:**
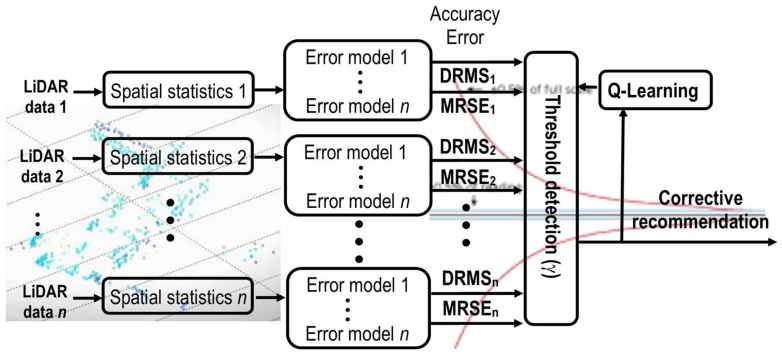
Flow diagram of the self-tuning method for the IoT sensor dynamic obstacle detection scenario.

The next step was to associate the same model of the error prediction model library to each LiDAR sensor. The conceptual diagram of the self-tuning method is shown in Figure 7.

During simulations of each scenario, the information provided by each sensor was collected, filtered and processed (as discussed in Section 3.1.1), the spatial statistics were calculated (as described in Section 3.1.2) and applied as inputs to each model in the library of error prediction models. These models estimate the accuracy of the localization for an object using the LiDAR sensors, based on the DMRS and MRSE figures of merits. Table 5 lists the value of the correlation (R^2^) of each type of model (ANN, *k*NN, and regression models) according to the number of LiDARs (1, 3, or 5) used at each instant with the objective of expanding the field of view, both vertical and horizontal, of the IoT LiDAR network in different critical situations. It must be stressed that the IoT sensors could not exchange sensory information without a local pre-processing phase in each IoT node. In this case, the shared information is the predicted error values obtained from each cloud point given by each sensor rather than raw sensory information.

From this table, the type of model within the library that showed a better correlation for outputs of both the 2D and the 3D spatial error types, in comparison with the different IoT LiDAR system configurations, could be extracted. With one LiDAR, the correlation values for both outputs were similar to those obtained in Section 3.1.3. On the other hand, for a configuration of three sensors, the value of this performance index improved notably in all models, highlighting *k*NN, as it is very close to 100%. Although, in theory, an increased number of sensors will widen the field of view, it turned out that the correlation with five sensors decreased in all models. One of the main causes is the duplicity of information provided by too many sensors. This behavior is also reflected in Table 5 where accuracy can be seen to increase with the number of LiDAR sensors. However, the accuracy also diminished when many LiDAR sensors brought too much sensory information. A problem could be solved by using an optimized mesh distribution that avoids the duplication of space covered by each LiDAR sensor.

**Figure 8:**
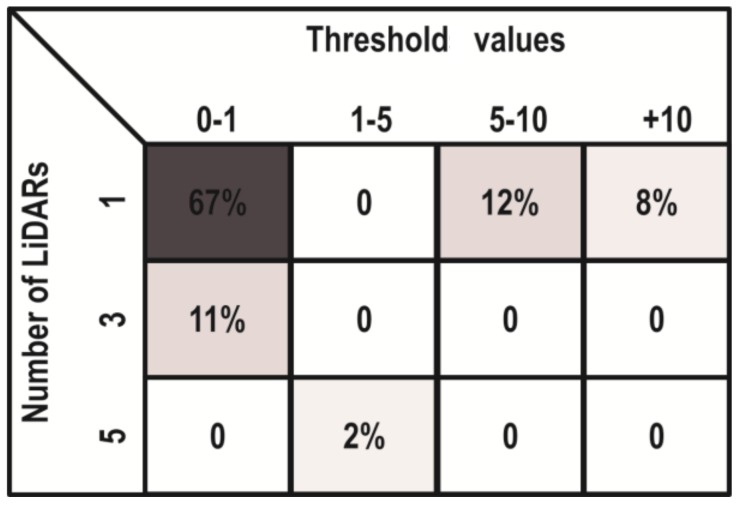
Q-learning classification error matrix.

Finally, during the simulation of this dynamic obstacle localization scenario, the learning and self-tuning (knowledge-based learning algorithm) tasks were also validated to automatically set the best prediction model and optimal number of LiDAR sensors needed to ensure reliability. The Q-learning classification error matrix is shown in the Figure 8.

As shown above, 67% of the scenarios can be appropriately addressed with a threshold between 0–1 (in other words, at a detection accuracy of over 99%) and only one LiDAR is needed. In total, 87% of the scenarios can be solved using only one LiDAR, but if the threshold is larger than 10 (with less than 90% of obstacle localization accuracy), then the use of three LiDARs is recommended, considerably increasing the reliability of the multi-sensor-based system. Furthermore, it is worth clarifying that only in 2% of cases are five LiDAR sensors needed, which is evidence of how the self-tuning method can minimize the number of sensors required to achieve higher obstacle localization reliability in the driving-assistance environments. In addition, the use of a minimal number of LiDAR sensors that are simultaneously required for reliable and accurate prediction may be highlighted as an important contribution. This rationalization of sensor use lowers energy consumption and saves on computing resources. Therefore, the Q-learning method that processes the prediction model error values at each instant can determine the minimum number of active LiDAR sensors that are needed, in order to ensure better reliability. It is only when this reliability cannot be assured with any distribution of LiDAR sensors that the activation of the stereoscopic camera is enabled.

## 5. Conclusions

The design of a self-tuning method and its implementation for automatic selection of the number of LiDAR sensors that are required to ensure object detection reliability in a IoT multisensory driving-assistance scenario has been presented in this paper. Three simple techniques from the perspective of industrial informatics have been considered in the implementation of the modeling library. A linear regression method has demonstrated the direct correlation between the extracted point clouds with obstacle localization. Secondly, the well-known multi-layer perceptron has once again been confirmed as a suitable technique for modelling the main process characteristics; and a *k*-nearest neighbors method has confirmed the suitability of clustering techniques to establish correlations based on point dispersions. All the selected models have accurately reflected the behavior of the selected variables and the statistical tests have confirmed the goodness-of-fit, highlighting that the *k*NN algorithm obtains a correlation coefficient of over 90% in almost all the scenarios.

Additionally, a Q-learning strategy was also introduced, in order to minimize the number of LiDAR sensors needed in each obstacle localization scenario and to ensure sensor reliability based on global IoT sensor network information. The self-tuning procedure that has been explored for each particular scenario has shown how many sensors are required for accurate obstacle localization in each scan. Based on those results, the proposed method has fulfilled two main criteria: the best model-based fitting and self-tuning management of the computational resources (smaller number of LiDAR real required for each particular situation) necessary for improvements in obstacle localization reliability on IoT LiDAR sensor networks. The accuracy and generalization of the proposed method, developed in a virtual driving traffic scenario with a Webots Automobile simulation tool, has solved 67% of the scenarios with one LiDAR with an obstacle localization accuracy of over 99%. Finally, the embedding of the proposed self-tuning methodology for validation in real driving environments is foreseen in future contributions to the European IoSENSE project (www.iosense.eu).

## Figures and Tables

**Table 1:** Q-learning reward matrix for detection threshold.

Detection Threshold (*γ*) Ranges	Rewards for Number of LiDARs		
	1	3	5
0–1	1.0	0.9	0.85
1–5	0.7	0.6	0.5
5–10	0.35	0.3	0.25
+10	0.15	0.1	0

**Table 2:** Specifications and location of both sensors on the vehicle.

Specifications	Ibeo Lux 4 Layers	Specifications	Bumblebee 2 1394a
Localization	Bottom frontal	Localization	Front top
Horizontal field	120 deg. (35 to −50 deg.)	Size resolution max.	1034 × 776 pixels
Horizontal step	0.125 deg.	Pixel resolution	4.65 µm square pixels
Vertical field	3.2 deg.	Focal lengths	3.8 mm
Vertical step	0.8 deg.	Aperture	Focal length/2.0
Range	200 m	Horizontal Field of View	66°
Update frequency	12.5 Hz	Frame rates	20 FPS

**Table 3:** Model correlation coefficients based on plane and space figures of merits.

Models	Correlation Coefficient (R^2^)	
	DMRS	MRSE
MLP	0.8668	0.8670
*kNN*	0.9355	0.9355
Linear Regression	0.4841	0.4858

**Table 4:** Localization of each sensor that comprises the IoT sensory system.

Sensor	Model	Localization (*m*)
3D Stereo Camera	Bumblebee 2	(0.0, 2.04, 1.2)
LiDAR 0	Ibeo Lux 4 layers	(0.0, 3.635, 0.5)
LiDAR 1	Ibeo Lux 4 layers	(−0.70, 3.64, 0.5)
LiDAR 2	Ibeo Lux 4 layers	(0.70, 3.64, 0.5)
LiDAR 3	Ibeo Lux 4 layers	(−0.55, 2.04, 1.2)
LiDAR 4	Ibeo Lux 4 layers	(0.55, 2.04, 1.2)

**Table 5:** Behavior of the correlation (R^2^) of each type of model according to the number of LiDAR sensors used at any one given time.

Techniques	Model Correlation (R^2^)					
	1 LiDAR		3 LiDAR		5 LiDAR	
	DRMS	MRSE	DRMS	MRSE	DRMS	MRSE
ANN	0.8190	0.8185	0.8949	0.9167	0.8263	0.8279
*kNN*	0.8184	0.8219	0.9868	0.9871	0.8893	0.8909
Regression	0.7317	0.7300	0.7572	0.7614	0.7269	0.7307
